# Diabetes Mellitus and Cardiomyopathy as Presenting Features of Occult Malignant Pheochromocytoma

**DOI:** 10.7759/cureus.19928

**Published:** 2021-11-26

**Authors:** Piti Inthaphan, Worapaka Manosroi, Puwitch Charoenchue, Komson Wannasai

**Affiliations:** 1 Division of Endocrinology, Department of Internal Medicine, Faculty of Medicine, Chiang Mai University, Chiang Mai, THA; 2 Department of Internal Medicine, Nakornping Hospital, Chiang Mai, THA; 3 Department of Radiology, Faculty of Medicine, Chiang Mai University, Chiang Mai, THA; 4 Department of Pathology, Faculty of Medicine, Chiang Mai University, Chiang Mai, THA

**Keywords:** reversible diabetes, occult malignancy, reversible cardiomyopathy, diabetes mellitus, adrenal pheochromocytoma

## Abstract

The concomitant occurrence of diabetes mellitus and cardiomyopathy secondary to occult malignant pheochromocytoma has rarely been reported. This case report describes the case of a 48-year-old female with a previous history of diabetes mellitus, hypertension, and cardiomyopathy who presented with fatigue and significant weight loss. Neither typical symptoms of pheochromocytoma nor metastatic symptoms were presented. Pheochromocytoma with extension to the liver was incidentally found from computed tomography of the abdomen and laboratory investigations during the work-up to identify the cause for the weight loss. Right adrenalectomy and a right hepatectomy were performed. Malignant pheochromocytoma was diagnosed based on pathology. All of her underlying conditions including diabetes mellitus, hypertension, and cardiomyopathy, were improved following the complete resection of the tumor. This case emphasizes the importance of early suspicion and diagnosis of malignant pheochromocytoma in individuals with atypical presentation of a chromaffin-secreting tumor.

## Introduction

Pheochromocytoma is a rare catecholamine-secreting neuroendocrine tumor that arises from chromaffin cells of the adrenal medulla [1]. Typical clinical manifestations of pheochromocytoma include episodes of headache, sweating, palpitation, and paroxysmal or sustained hypertension. Most patients are symptomatic, while approximately 25% are asymptomatic or have atypical presentations such as dilated cardiomyopathy, abdominal pain, respiratory distress, myocardial infarction, pulmonary edema, hyperthermia, or cardiogenic shock [2]. Pheochromocytoma-induced cardiomyopathy is a rare form of presentation. Due to its uncommon nature, there have been no published reports regarding its incidence. Glucose intolerance has been documented in pheochromocytoma patients together with other symptoms in up to 50% of cases, [3] whereas diabetes mellitus has been reported in 23% to 33% of large symptomatic pheochromocytoma patients [4,5]. Intriguingly, total resection of pheochromocytoma can reverse or lead to the improvement of these presentations [6, 7]. 

According to the new criteria from WHO, malignant pheochromocytoma is defined as frank locoregional invasion or distant metastasis from the primary neoplasm of a chromaffin tumor [8]. Currently, histological criteria for a diagnosis of malignant pheochromocytoma are seldom employed. Malignant pheochromocytoma is only infrequently reported as a clinically silent tumor. Most of the case reports have been presented with typical symptoms of pheochromocytoma or metastatic symptoms [9, 10].

To date, diabetes mellitus and cardiomyopathy as first presentations in occult malignant pheochromocytoma have been rarely reported. The present case describes a 48-year-old patient who presented with diabetes mellitus and cardiomyopathy without the typical triad of pheochromocytoma (headache, palpitation, and sweating) for a period of 2 years prior to the diagnosis of occult malignant pheochromocytoma. 

## Case presentation

A 48-year-old female diagnosed with diabetes mellitus, hypertension, and dilated cardiomyopathy for two years presented with fatigue and significant weight loss of 5 kilograms within one month. She was treated with enalapril 10 mg/day, premixed insulin 40 unit/day, and metformin 2000 mg/day. 

**Figure 1:**
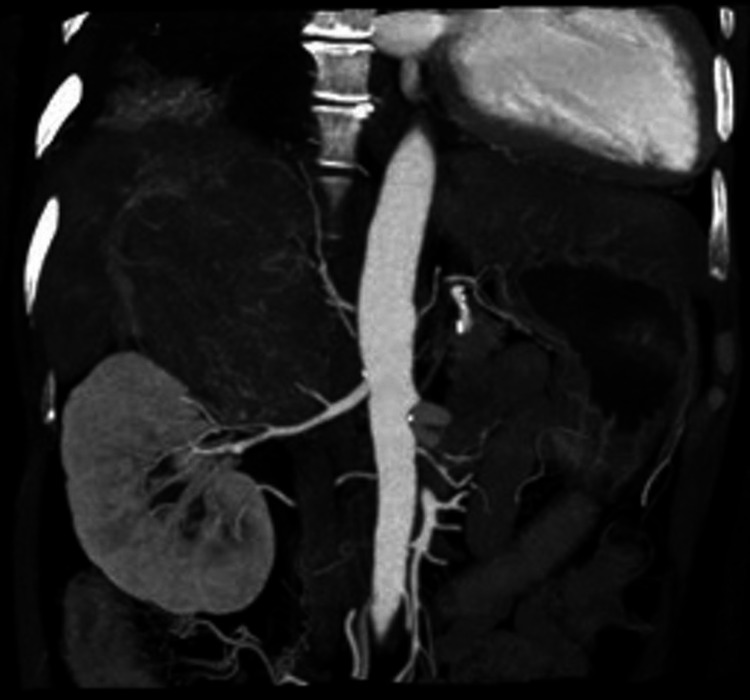
CT whole abdomen coronal view with contrast media

Computed tomography (CT) of the abdomen was performed during the workup to determine the cause of the significant weight loss. The CT of the abdomen revealed a 6.4x6.9x9.8 cm enhanced arterial phase mass with contrast washout on the venous and delayed phase of the right adrenal gland with hepatic involvement at the posterior right lobe segments 6 and 7) (Figure 1).

CT-guided core-needle biopsy and fine-needle aspiration biopsy were performed. Pathology examination found a well-differentiated metastatic neuroendocrine tumor (NET), G2, immunohistochemistry positive for chromogranin A and synaptophysin. 

**Figure 2:**
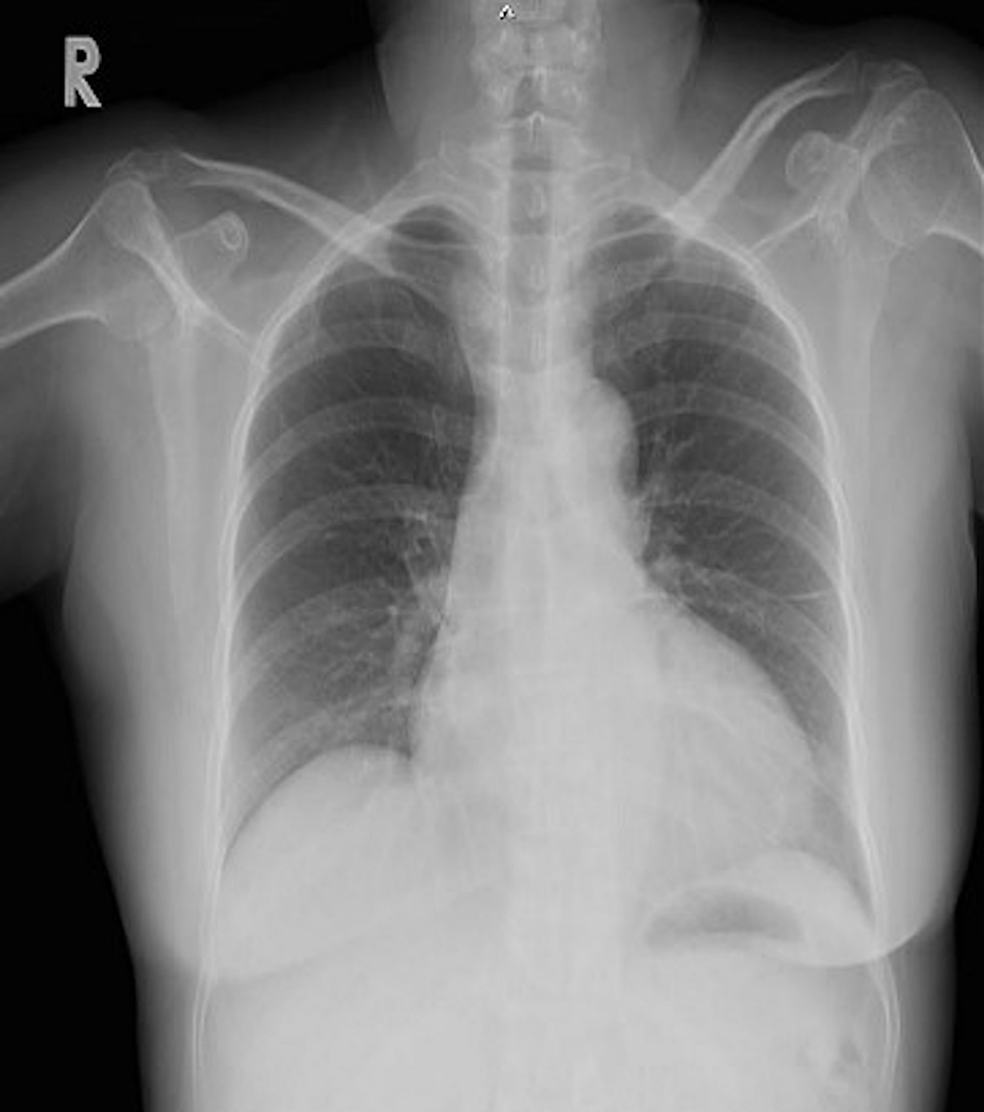
Chest x-ray (posteroanterior view)

She was then transferred to an endocrinologist for evaluation of the right adrenal mass. Additional medical history revealed that she had had easy fatigability and an unintentional weight loss of 3 kg within the previous month. She denied a history of headaches, palpitation, or sweating. She had dyspnea without edema, particularly in the supine position and on exertion. There was no known history of pheochromocytoma, cancer, or diabetes mellitus in her family. Upon physical examination, she was found to be slightly dehydrated. Her height was 154 cm, weight 51 kg, and BMI 21.5 kg/m2. Her highest blood pressure (BP) was 160/102 mmHg. No acanthosis nigricans was seen. Lungs were clear on auscultation. Heart rate was slightly elevated at 116 beats/min and regular. Her point of maximal impulse was identified at the 6th intercostal space just anterior to the anterior axillary line. On laboratory investigation, fasting blood glucose was elevated at 9.5 mmol/L and HbA1C was elevated at 7.6%. Her kidney function was normal. Her electrocardiogram showed sinus tachycardia, left atrial enlargement, and left ventricular hypertrophy without evidence of ischemia or infarction. Chest x-ray showed cardiomegaly without pulmonary congestion (Figure 2).

Echocardiography revealed global hypokinesia with left ventricular ejection fraction (LVEF) 28% which suggested cardiomyopathy. Assessments of the functional status of the adrenal mass were performed. The 24-hr urine metanephrine and normetanephrine were significantly elevated (Table 1).

**Table 1:** Adrenal mass functional status

Tests	Patient value	Normal value
24-hr urine metanephrine	2,623.8 µg/day	25-312 µg/day
24-hr urine normetanephrine	8,533.3 µg/day	35-445 µg/day
Serum cortisol after 1 mg overnight dexamethasone suppression	0.7 µg/dL	<1.8 µg/dL

Differential diagnosis

The patient was provisionally diagnosed with right pheochromocytoma leading to secondary diabetes mellitus, hypertension, and cardiomyopathy. The differential diagnosis included a large right adrenal mass extending to the liver comprised of malignant pheochromocytoma and adrenocortical carcinoma. Based on the elevated levels of urine metanephrine and normetanephrine, the most plausible diagnosis was malignant pheochromocytoma. As mentioned previously, malignant pheochromocytoma is defined as locoregional invasion or distant metastasis from the primary neoplasm of a chromaffin tumor, and histological criteria are infrequently employed. Regarding the cause of her underlying diseases (diabetes mellitus, hypertension, and cardiomyopathy), the differential diagnosis, other than secondary to pheochromocytoma, was metabolic syndrome with long-lasting hypertension. If these underlying conditions were improved or resolved after removal of the pheochromocytoma, it could be presumed that the pheochromocytoma was the leading cause of those underlying conditions.

Treatment

The patient underwent an open right adrenalectomy, extended right hepatectomy, and cholecystectomy along with inferior vena cava (IVC) reconstruction due to the extension of the mass to the right lobe of the liver. Perioperative management for pheochromocytoma was properly performed, including appropriate blood pressure control with an alpha-blocker and additional metabolic profile control and monitoring during the perioperative period.

Outcome and follow-up

**Figure 3:**
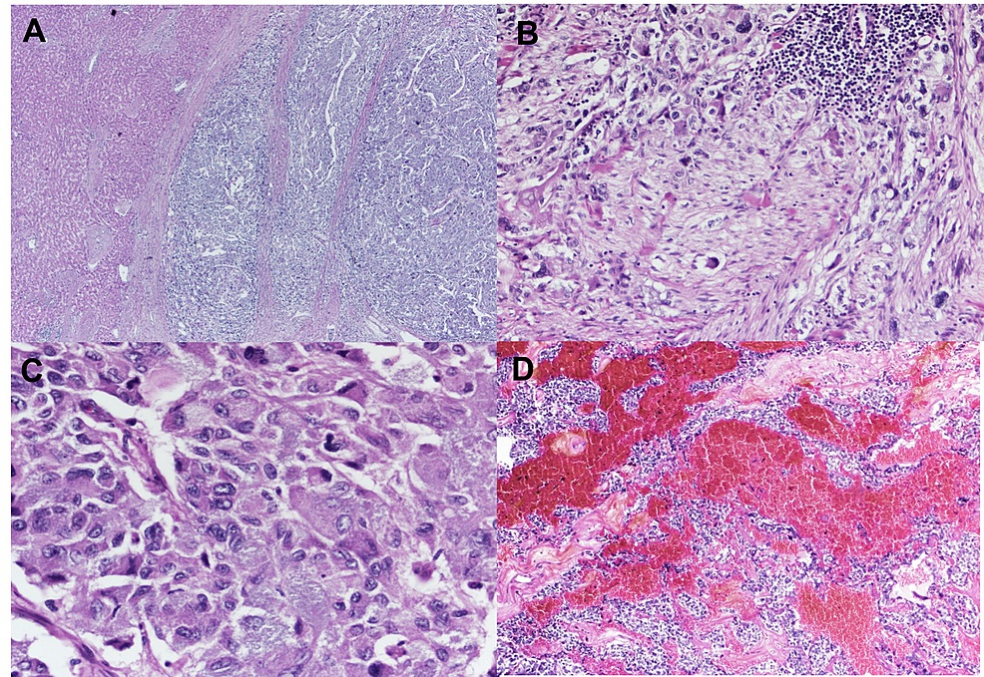
Microscopic examination of right adrenal gland. (A) Mitotic counts were noted (H&E 400x), (B) A section showing hemorrhage within the tumor (H&E 100x), (C) Tumor cells invading the hepatic capsule (H&E 100x), and (D) the nerve (H&E 200x).

The resected tumor from the right adrenal gland measured 9x8x7 cm. Microscopic examination of the right adrenal mass showed nested tumor cells (zellballen) with a large and polygonal appearance. The tumor cells contained abundant fine granular red-purple cytoplasm with pleomorphism of the nuclei and prominent nucleoli (Figure 3).

At the 1-month postoperative follow-up, 24-hr urine metanephrine and normetanephrine had decreased to 332.5 and 549.79 mcg, respectively. At the 4-month postoperative follow-up, 24-hr urine metanephrine and normetanephrine tests were within the normal range, and the enhancing mass at the right adrenal gland was no longer visible in the CT scan of the abdomen. After the surgery, the patient’s BP remained normal without any anti-hypertensive medication. She reported no pre-syncopal or syncopal episodes after surgery. Her fasting blood glucose was well controlled at 121 to 133 mg/dL, and HbA1C was 6.44% with metformin of only 1,000 mg/day. The dose of premixed insulin was gradually reduced and could be completely discontinued. Her dyspnea and fatigue also gradually improved. Postoperative echocardiography revealed an LVEF of 44%. As there was an improvement in her underlying conditions, it could be confirmed that the resected malignant pheochromocytoma was the main culprit.

## Discussion

In this case, we present a patient with difficult-to-control diabetes mellitus, hypertension, and dilated cardiomyopathy resulting from occult malignant pheochromocytoma. The supporting hypothesis that diabetes mellitus and cardiomyopathy were the results of malignant pheochromocytoma was that their onset occurred before the pheochromocytoma was identified and improved after pheochromocytoma has been resected. Interestingly, neither typical presentations of pheochromocytoma nor malignant symptoms of pheochromocytoma were presented in this patient.

The pathogenesis underpinning hyperglycemia or diabetes mellitus in pheochromocytoma involves multifactorial causes. Catecholamines compromise insulin secretion and glucose utilization via beta-adrenoreceptor desensitization, increased lipolysis, and an enhanced pro-inflammatory state which decreases hepatic insulin sensitivity [11]. Ectopic somatostatin, adrenocorticotropic hormone (ACTH), or corticotropin-releasing hormone (CRH) secreting-pheochromocytoma are additional mechanisms that can lead to glucose intolerance and/or diabetes [12]. Increased levels of hormonal secretion from large tumors are likely to impair glucose tolerance. In a previous study, Beninato et al. stated that patients with tumors larger than 4.7 cm were more likely to have diabetes and were associated with significantly higher catecholamine levels than those with smaller tumors [4]. Other reported factors which can increase the risk of diabetes in pheochromocytoma include older age and elevated BMI [13]. On the other hand, increasing BMI as a risk factor for diabetes may be concealed by catecholamine-induced lipolysis causing weight loss in some patients [14].

Difficulty in achieving glycemic control with standard diabetes therapy or the occurrence of diabetes in lean hypertensive patients could be important cues to seek for occult pheochromocytoma [5,7]. Pheochromocytoma removal is more effective than an alpha-blocker for restoring normal glucose and insulin homeostasis and usually leads to the resolution of diabetes if there are no other contributing factors [4]. Diabetes mellitus has been reported to have been cured in up to 70-90% of cases between the immediate postoperative period and the 1-year follow-up [5]. However, the resolution period for diabetes improvement postoperatively has not yet been definitively determined and can vary among patients.

Dilated cardiomyopathy is an unusual cardiovascular manifestation of pheochromocytoma [15]. Only 4% of pheochromocytoma-induced cardiomyopathy patients were reported to have had the classical pheochromocytoma triad of palpitations, headache, and diaphoresis [6]. Catecholamine excess can result in functional hypoxia-induced myocardium damage from increased contractility and coronary spasms which can lead to decreased blood flow, increased oxygen consumption due to excessive free fatty acid-induced mitochondrial uncoupling, excess intracellular calcium, and generation of oxidative stress [16]. One report stated that pheochromocytoma resection led to an improvement of cardiomyopathy in 96% of patients whereas 44% of cases without resection were associated with death or cardiac transplantation [6]. Resolution of cardiomyopathy following pheochromocytoma resection occurred between 6 weeks and 16 months in reported cases [17,18].

To the best of our knowledge, there have been few English language case reports of the co-occurrence of dilated cardiomyopathy and diabetes mellitus in pheochromocytoma patients [19, 20]. Characteristics of each of those patients are shown in Table 2.

**Table 2:** Case reports of diabetic patients with chronic pheochromocytoma and cardiomyopathy

Patient sex, age (years)	Pheochromocytoma location (size)	Duration of diabetes mellitus / cardiomyopathy before pheochromocytoma diagnosis	Presenting symptoms	Reference number
Female, 47	Right adrenal gland (7x7 cm)	8 years / 8 years	Dyspnea from heart failure	[7]
Female, 45	Right adrenal gland (5 cm)	N/A / 5 years	Constipation alternating with diarrhea, left iliac fossa pain (ischemic sigmoid colitis)	[20]

Unlike the present case, in each case, the pheochromocytomas were benign, unlike the present case which was malignant.

## Conclusions

The co-occurrence of diabetes mellitus and cardiomyopathy in malignant pheochromocytoma is considered unusual. One should have a high level of awareness of the potential need to screen for pheochromocytoma even in cases where there is no typical presentation of symptoms. Secondary causes of diabetes mellitus, e.g., pheochromocytoma, should be suspected when blood glucose levels are difficult to control, particularly when it occurs in combination with hypertension and idiopathic cardiomyopathy. Prompt detection of pheochromocytoma and early treatment will increase the likelihood of curing comorbidities and preventing additional complications. Furthermore, if the tumor is malignant, the earliest possible detection can increase the chances of satisfactory results from the surgery. Lifelong surveillance for recurrence of pheochromocytoma and adjustment of medications to control other comorbidities should be initiated after pheochromocytoma removal. 
